# Correlation between *pri-miR-124* (rs531564) polymorphism and congenital heart disease susceptibility in Chinese population at two different altitudes: a case-control and *in silico* study

**DOI:** 10.1007/s11356-019-05350-4

**Published:** 2019-05-29

**Authors:** Wenke Yang, Kang Yi, Hongmiao Yu, Yunhan Ding, Dehong Li, Yuping Wei, Tao You, Xiaodong Xie

**Affiliations:** 1 School of Basic Medical Science, Lanzhou University, Lanzhou, 730000 China; 2 Gansu Cardiovascular Institute, People’s Hospital of Lanzhou City, Lanzhou, 730050 China; 3 Department of Cardiac Surgery, Gansu Provincial Hospital, Lanzhou, 730000 China; 4 Congenital Heart Disease Diagnosis and Treatment Gansu Province International Science and Technology Cooperation Base, Lanzhou, 730000 China

**Keywords:** MiR-124, Single nucleotide polymorphism, Congenital heart disease, Hypoxia, Altitude

## Abstract

The development of congenital heart disease (CHD) is a complicated process and affected by multiple environmental factors, as genetic factors, and the interactions among those factors. Previous studies have shown that intrauterine hypoxic environment exposure is a risk factor of CHD, but the genetic factors involved in the process are not clear. In this study, given that tetralogy of Fallot (TOF) is a CHD with hypoxemia as its primary pathophysiological manifestation, an *in silico* analysis was performed to reveal the relationship between potential target genes (miR-124) with the energy metabolism in non-syndromic TOF patients’ cardiomyocyte. Furthermore, the study investigated the correlation between the primary miR-124 (rs531564) polymorphism and CHD susceptibility in 432 sporadic patients and 450 controls from two different altitude provinces (city) in China. Our study indicated that the minor C allele of rs531564 correlated with reduced risk of CHD in the low altitude city. Besides, the C allele has elevated frequency in the high-altitude group. Therefore, our findings suggest that the minor C allele of rs531564 SNP may be involved in the reduction of the risk of CHD in a way that interacts with the intrauterine hypoxic environmental factors.

**Electronic supplementary material:**

The online version of this article (10.1007/s11356-019-05350-4) contains supplementary material, which is available to authorized users.

## Introduction

Congenital heart disease (CHD) is the most prevalent human birth malformation with an incidence of 1 approximately in 100 newborns. CHD is also a major cause of perinatal and infant mortality, with about 220,000 deaths worldwide each year (Marelli et al. 2014). The exact etiology of CHD is not fully understood, especially the etiology of sporadic, non-syndromic CHD. Previous studies have shown that multiple factors contribute to the risk of CHD in various geographic areas and genetic backgrounds, suggesting the complex mechanisms underlying the development of CHD, which involve numerous environmental and genetic factors, and also, the interaction between them (Hinton 2013). Intrauterine hypoxic environment exposure, caused by high-altitude hypoxic environment or placental insufficiency, is one of the risk factors for CHD (Bae et al. 2003; Gagnon 2003; Hasan 2016; Herrera et al. 2016; Miao et al. 1988; Yue and Tomanek 1999; Zheng et al. 2013). The possible molecular mechanism might be associated with the fact that chronic fetal hypoxia alters cardiac gene expression, accelerates pre-existing cardiomyocytes exit the cell cycle, increases myocyte apoptosis, and decreases in the number of cardiomyocytes (Botting et al. 2014; Osterman et al. 2015; Zhang 2005).

Recent studies have reported that several microRNAs (miRNAs) play pivotal roles in cardiac development under hypoxic intrauterine environment, being termed as hypoxia-responsive miRNAs (Azzouzi et al. 2015; Lock et al. 2017). Such miRNAs, as miR-199a and miR-210, presented a significant correlation with cardiac energy metabolism. Rane et al. 2009 suggested that miR-199a, is a miRNA downregulated under hypoxia, regulated cardiac metabolism by targetting hypoxia-inducible factor-1 alpha and sirtuin 1. Hu et al. 2010 reported that upregulated hypoxia-responsive miR-210 exhibits a cardio-protective effect by inhibiting *Efna3* and *Ptp1b*, in a mouse model of myocardial infarction. Besides, it is widely reported that miR-124 is a downregulated hypoxia-responsive miRNA involved in the regulation of biological processes in a variety of cells (Gong et al. 2017; Gu et al. 2016; Li et al. 2017). Moreover, a line of evidence has demonstrated that rs531564 (G>C), a functional SNP of the primary miR-124 (*pri-miR-124*) sequence, is associated with the expression of mature miR-124. The G allele carriers, which represent the major population, have a higher expression level of miR-124 transcript than the C allele carriers, which represent a minor population (Chen et al. 2018; Li et al. 2017; Zou et al. 2017). The rs531564 polymorphism of *pri-miR-124* is also one of the risk factors for human cervical cancer, esophageal squamous cell carcinoma, colorectal cancer, and type 2 diabetes (Gao et al. 2015; Li et al. 2015; Wu and Zhang 2014; Ye et al. 2008). The downregulated miR-124 also exhibits a cardio-protective function by attenuating endoplasmic reticulum stress (Bao et al. 2017). However, evidence supporting a correlation between the rs531564 polymorphism of *pri-miR-124* and CHD is inadequate.

Tetralogy of Fallot (TOF) is one of the most common forms of cyanotic CHD. The main pathological manifestation of this type of CHD is chronic hypoxemia (Apitz et al. 2009). In the present study, gene expression and miRNA expression profiles pertaining to non-syndromic TOF were downloaded from the Gene Expression Omnibus database (Clough and Barrett 2016). These datasets were utilized to identify key genes and miRNAs that play significant roles in various biological processes in cardiomyocytes under hypoxic condition by running through three target gene prediction databases, TargetScan (Agarwal et al. 2015), miRTarBase (Chou et al. 2018), and RNA22 (Miranda et al. 2006). The results indicated the correlation between the energy metabolism in cardiomyocytes of non-syndromic TOF and the downregulation of miR-124 and several other potential target genes. We also investigated the correlation between the rs531564 polymorphism of *pri-miR-124* and CHD in two groups of patients from an area with different altitudes. One group is from Beijing (altitudes ranging from 28 to 96 m); the other group is from Gansu (altitudes ranging from 1085 to 2690 m). The study also indicated that the minor C allele of rs531564 polymorphism of *pri-miR-124* was associated with a reduced risk of CHD in Beijing population but not Gansu population.

## Methods

### Microarray data

Two gene expression profiles (GSE26125 and GSE35776) and a miRNA expression profile of GSE35490 were obtained from the GEO database. The GSE26125 dataset, based on the platform of GPL11329 (Applied CodeLink Human Whole Genome Bioarray), consisted of samples from 16 idiopathic TOF patients without the 22q11.2 deletion and five controls. The GSE35776 dataset, based on the platform of GPL5175 (Affymetrix Human Exon 1.0 ST Array), consisted of samples from 16 non-syndromic TOF patients without the 22q11.2 deletion, three fetal hearts, and eight normal controls. The miRNA expression profile of GSE35490, based on the platform of GPL8786 (Affymetrix Multispecies miRNA-1 Array), shared the same sample source as GSE35776. A more detailed description of the included samples can be found in two previously published studies (Bittel et al. 2011; O’Brien et al. 2012).

### Data processing and *in silico* analysis

The *limma* package in R software was applied to perform data preprocessing and screening of differentially expressed genes (DEGs) as well as miRNAs (DEMIs) (Ritchie et al. 2015). For each dataset, DEGs or DEMIs between non-syndromic TOFs and normal controls were screened. The threshold values for the screening include the following: |log_2_ fold change (FC)| > 1.00 and *P* < 0.05. The overlap of DEGs between GSE26125 and GSE35776 was used for subsequent analysis. In the present study, three online prediction programs (TargetScan, miRTarBase, RNA22) were used for screening the potential targets of DEMIs. The overlapped portion of the results from the three online prediction programs was considered as the potential targets of DEMIs.

Gene Ontology (GO) term enrichment and Kyoto Encyclopedia of Genes and Genomes (KEGG) pathway enrichment analysis of the identified DEGs were performed using KOBAS 3.0, a most recently updated online tool for gene function annotation (Xie et al. 2011). Significant GO terms and KEGG pathways enriched with upregulated or downregulated DEGs were screened with a criterion of *P* < 0.05.

To explore the interactive relationships among DEGs, a protein-protein interaction network was constructed by the Search Tool for the Retrieval of Interacting Genes (STRING, version 10.0), and a combined score of 0.40 was set as the cut-off criterion (Szklarczyk et al. 2017). After filtering out target genes of DEMIs, the regulation relationships between the miRNA- mRNA and protein-protein interaction were integrated. The network was constructed using Cytoscape (www.cytoscape.org). The key gene modules were identified from PPI network by using the Molecular Complex Detection (MCODE) plugin based on Cytoscape. The number of nodes was equal or more than 5; a MCODE score more than 5 was set as cut-off criterion. The pathway enrichment analysis of genes in the modules was performed with KOBAS 3.0. *P* < 0.05 was considered to be significant.

### Gene set enrichment analysis

The importance of Gene Set Enrichment Analysis (GSEA) is its capability to capture the correlation between weakly differential expression in gene sets and phenotypes (Subramanian et al. 2005). GSEA was able to not only verify the DEGs enrichment analysis but also cover up the shortcomings of DEGs analysis. In the present study, GSEA was used to compare the expression profiles of gene sets named h.all.v6.1.symbols.gmt [Hallmarks], which is from Molecular Signature Database, between non-syndromic TOFs and normal controls. The gene expression profiles that were analyzed by GSEA were GSE26125 and GSE35776. Normalization enrichment score (NES), *P* value, and false discovery rate (FDR) were calculated. A gene set is considered significantly enriched with the criteria of *P* < 0.05 and FDR < 0.25. GSEA was performed by using the GSEA 3.0 program, provided by Broad Institute (http://www.broadinstitute.org/gsea/index.jsp).

### Samples

Blood samples were obtained from 2 independent case-control groups. In total, 432 CHD patients and 450 healthy controls were recruited in this study. The Beijing group consisted of 120 CHD patients and 130 healthy controls. The Gansu group was composed of 312 CHD patients and 320 healthy controls. Sporadic CHD patients underwent chest X-ray examination, electrocardiography, and echocardiography. Patients who were diagnosed with CHD but without recognized genetic syndrome, chromosome abnormalities, or family history of CHD were enrolled in this study. The controls were recruited from the same geographical area. They were age, gender, and ethic matched with unrelated subjects enrolled in the present study and from the same hospital for health screening. The study was approved by the Ethics Committees of Lanzhou University of Basic Medical Science (20160204) and all participants provided informed consent forms.

### DNA extraction and genotyping

Genomic DNA was extracted from peripheral blood using a Cwbio® Blood Genomic DNA Mini Kit (Cwbio, Beijing, China). SNP genotyping was performed by Shanghai Genesky Bio-Tech Genetic Core Lab (Shanghai, China) using multiplex ligation detection reaction. Approximately 5% of the samples were randomly selected to validate the genotyping results by directly sequencing and the reproducibility of the genotyping results was 100%. The primers used for PCR and DNA sequencing were as follows:

Forward: 5′-TCTTCTACCCACCCCTCTTCC-3′;

Reverse: 5′-AATCTGCACACACAAGCACTC-3′.

### Statistical analysis

The Hardy-Weinberg equilibrium (HWE) of the genotype distributions of the controls was determined by a goodness-of-fit chi-square (*χ*2) test. Continuous and categorical variables were estimated by Student’s *t* test and the *χ*2 test, respectively. Allele and genotype frequency distributions of rs531564 in patients and controls were evaluated by *χ*2 tests. The relationship between rs531564 polymorphism and CHD risk was analyzed by unconditional logistic regression model, and the odds ratio (OR) and the 95% confidence intervals (95% CI) with adjustments for age and gender were calculated. All statistical analyses were performed with the SPSS19.0 software package (SPSS, Chicago, IL, USA) and *P* < 0.05 was considered statistically significant.

## Results

### The downregulated miR-124 was a potential key miRNA in non-syndromic TOF

Screened with the thresholds of |log2(FC)| > 1.00 and *P* < 0.05, 3211 and 641 upregulated DEGs and 498 and 265 downregulated DEGs were identified from GSE26125 and GSE35776, respectively (Fig. S1a, b). Three hundred eighteen upregulated DEGs and 12 downregulated DEGs were overlapped between the two gene expression profile (Fig. S1d, e) (Supplementary Table S1). In addition, a total of 49 DEMIs, 21 upregulated miRNAs, and 28 downregulated miRNAs were identified from GSE35490 (Fig. S1c). Only those miRNA target genes that also intersect with DEGs were selected. Finally, 29 pairs of potential miRNA-mRNA interactions were identified, including 10 miRNAs and 28 mRNAs. The downregulated miR-124 (log_2_(FC) = − 1.26, *P* < 0.001) was predicted to target 10 upregulated genes, including *CALU*, *AK3*, *ABHD5*, *LPCAT3*, *CHP1*, *MYPN*, *AK2*, *PGRMC2*, *ATP6V0E1*, and *ACAA2*.

The results from the GO analysis revealed that the upregulated DEGs were significantly enriched in biological processes (BP) that were involved in cellular (*P* = 2.09e−23), metabolic (*P* = 1.14e−20), and oxidation-reduction processes (*P* = 1.54e−20). Based on the KEGG pathway enrichment analysis, upregulated DEGs were significantly enriched in metabolic pathways (*P* = 7.96e−14), oxidative phosphorylation (*P* = 2.27e−09), and proteasome (*P* = 2.99e−07). The top 10 GO biological processes and KEGG pathways enriched for upregulated DEGs were presented in Table S2.

**Fig. 1:**
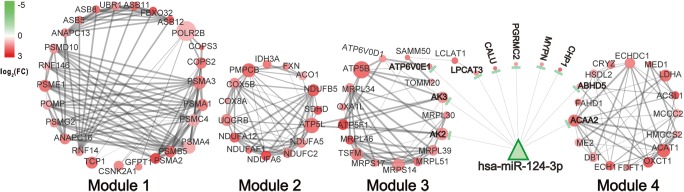
The key modules of protein-protein interaction network. The red cycles indicate upregulation DEGs, and the green triangle represents the downregulated miR-124. Size of the nodes indicates the interaction degrees

The PPI network of DEGs was comprised of 212 nodes and 584 edges. The top four modules the PPI networks involved in the process related to the proteasome, oxidative phosphorylation, metabolic pathways, cardiac muscle contraction, and peroxisome (Fig. 1) (Table S3). After integrating with 29 miRNA-DEG regulatory pairs, the predicted target genes of DEMI miR-124 were presented in both Module 3 and Module 4, which were enriched in the processes related to oxidative phosphorylation, metabolic pathways, as well as peroxisome (Fig. 1) (Table 1).

**Table 1:** KEGG pathway enrichment analysis of gene modules and DEMI-DEG regulatory relationships in gene modules

Module 3, score 6.500				
KEGG pathway	*P*	Cor. *P*	Node IDs	DEMI(DEG)
hsa00190 oxidative phosphorylation	3.48e−05	0.001	*TSFM*, *AK2*, *AK3*, *ATP5B*, *ATP5F1*, *MRPL30*, *MRPS17*, *MRPL34*, *MRPS14*, *MRPL46*	miR-124 (*AK2*, *AK3*)
hsa01100 metabolic pathways	0.003	0.010		
hsa00230 purine metabolism	0.004	0.010		
hsa03010 ribosome	6.36e−07	1.02e−05		
Module 4, score 6.333				
KEGG pathway	*P*	Cor. *P*	Node IDs	DEMI(DEG)
hsa01212 fatty acid metabolism	8.44e−05	0.001	*MCCC2*, *ECHDC1*, *HSDL2*, *ACAT1*, *OXCT1*, *ECH1*, *ACAA2*	miR-124 (*ACAA2*)
hsa01100 metabolic pathways	0.004	0.008		
hsa04146 peroxisome	0.023	0.025		

*Cor. P*, corrected *P* value

### Gene set enrichment analysis

**Fig. 2:**
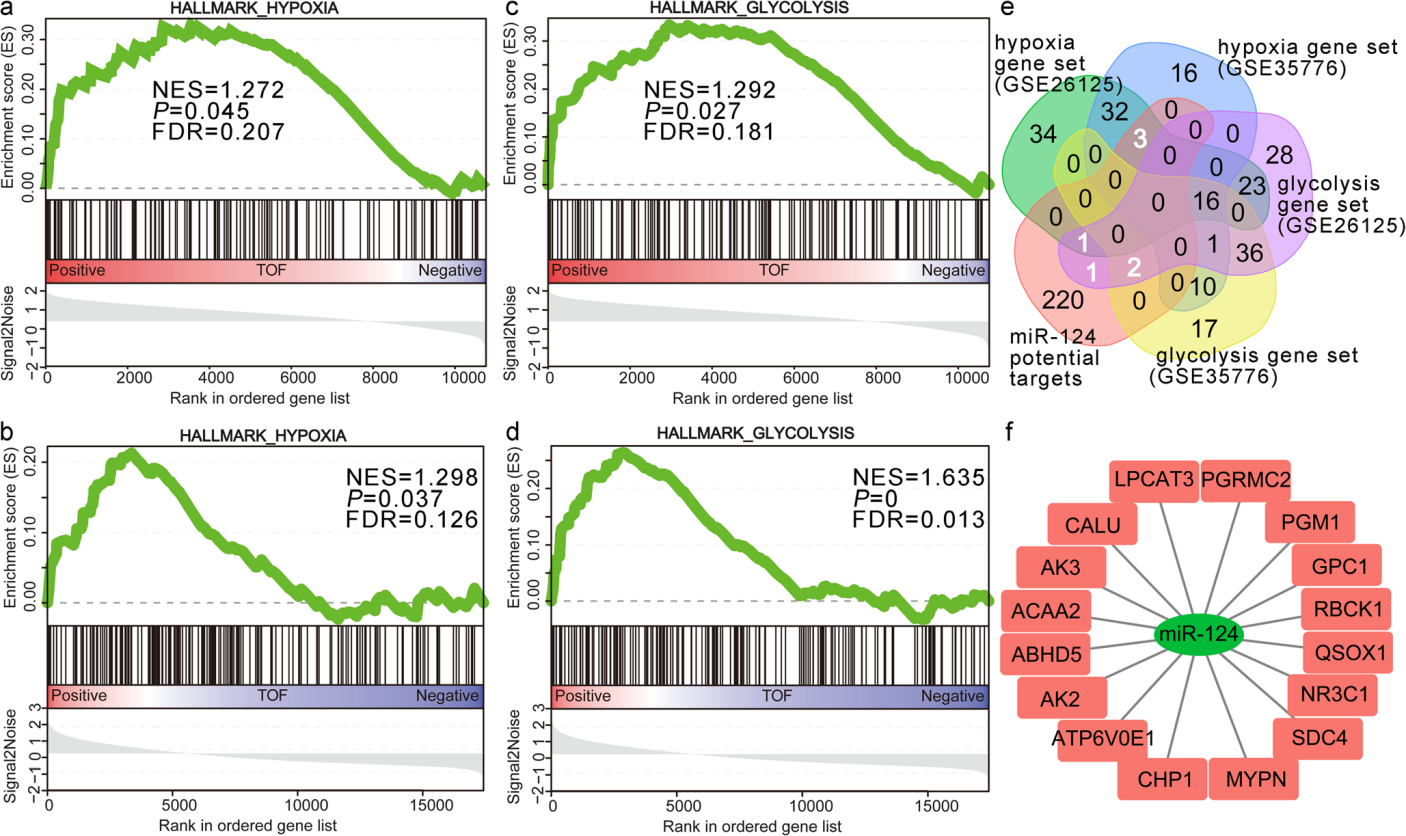
Gene set enrichment analysis in non-syndromic TOF. **a**–**d** Hypoxia- or glycolysis-related gene set was enriched in GSE26125 and GSE35776, respectively. NES, normalized enrichment score. FDR, false discovery rate; **e** Venn diagram illustrating the overlapped area that positively correlated with hypoxia, glycolysis gene sets, and miR-124 potential targets; **f** potential targets of miR-124

To further confirm DEGs enrichment analysis, GSEA was conducted in two independent non-syndromic TOF GEO datasets (GSE26125 and GSE35776). The results produced 13 upregulated gene sets from non-syndromic TOF compared with control and non-downregulated gene sets (Table S4). Metabolic pathways, oxidative phosphorylation, and peroxisome gene sets were significantly enriched. Moreover, we noticed that gene sets related to hypoxia (Fig. 2a, b), glycolysis (Fig. 2c, d), DNA repair, and mTORC1 signaling were also significantly enriched in non-syndromic TOF.

The result demonstrated that four potential target genes of miR-124 (*SDC4*, *PGM1*, *NR3C1*, and *GPC1*) were found in the hypoxia gene set and four potential target genes (*AK3*, *GLRX*, *RBCK1*, and *GPC1*) were listed in the glycolysis gene set (Fig. 2e). Combined with the target genes found from DEGs analysis, a total number of 16 potential target genes of miR-124 were identified in both of the non-syndromic TOF mRNA profiles, GSE26125 and GSE35776 (Fig. 2f).

### Characteristics of the study subjects

The characteristics of the study subjects are summarized in Table 2. The mean age of the CHD patients and controls was 9.53 ± 13.88 years and 9.48 ± 12.11 years, respectively. The gender distribution in the CHD patients was 234 males and 198 females, and there were 261 males and 189 females among the healthy controls. There was no statistical difference between the cases and the controls regarding age (*P* = 0.95) and gender (*P* = 0.25). Moreover, there was no statistical difference in terms of age and gender distribution between the cases and the controls in both the Beijing group and Gansu group.

**Table 2:** Clinical characteristics in CHD cases and controls

Variable	Cases		Controls		*P* value
	No.	%	No.	%	
Beijing group	120		130		
Age, years (mean ± SD)	2.50 ± 2.15		2.78 ± 2.21		0.31
Gender (male/female)	60/60	50/50	65/65	50/50	1.00
CHD classification					
Isolated/complex CHD^a^	57/63	47.5/52.5			
Detailed CHD phenotypes					
ASD/VSD/TOF/others	13/44/19/44				
Gansu group	312		320		
Age, years (mean ± SD)	12.22 ± 15.46		12.20 ± 13.38		0.98
Gender (male/female)	174/138	55.8/44.2	196/124	61.25/38.75	0.16
CHD classification					
Isolated/complex CHD^a^	226/86	72.4/27.6			
Detailed CHD phenotypes					
ASD/VSD/TOF/others	97/129/26/60				
Combined group	432		450		
Age, years (mean ± SD)	9.53 ± 13.88		9.48 ± 12.11		0.95
Gender (male/female)	234/198	54.2/45.8	261/189	58/42	0.25
CHD classification					
Isolated/complex CHD^a^	283/149	65.5/34.5			
Detailed CHD phenotypes					
ASD/VSD/TOF/others	110/173/45/104				

^a^Isolated CHD including ASD and VSD. Complex CHD including TOF, AVSD, TOF + ASD, ASD + PDA, VSD + PDA, ASD + PS, and VSD + PS

*CHD* congenital heart disease, *ASD* atrial septal defect, *VSD* ventricular septal defect, *TOF* tetralogy of Fallot, *AVSD* atrioventricular septal defect, *PDA* patent ductus arteriosus, PS pulmonary stenosis, *SD* standard deviation

### Correlation between the rs531564 SNP and the susceptibility to CHD

**Fig. 3:**
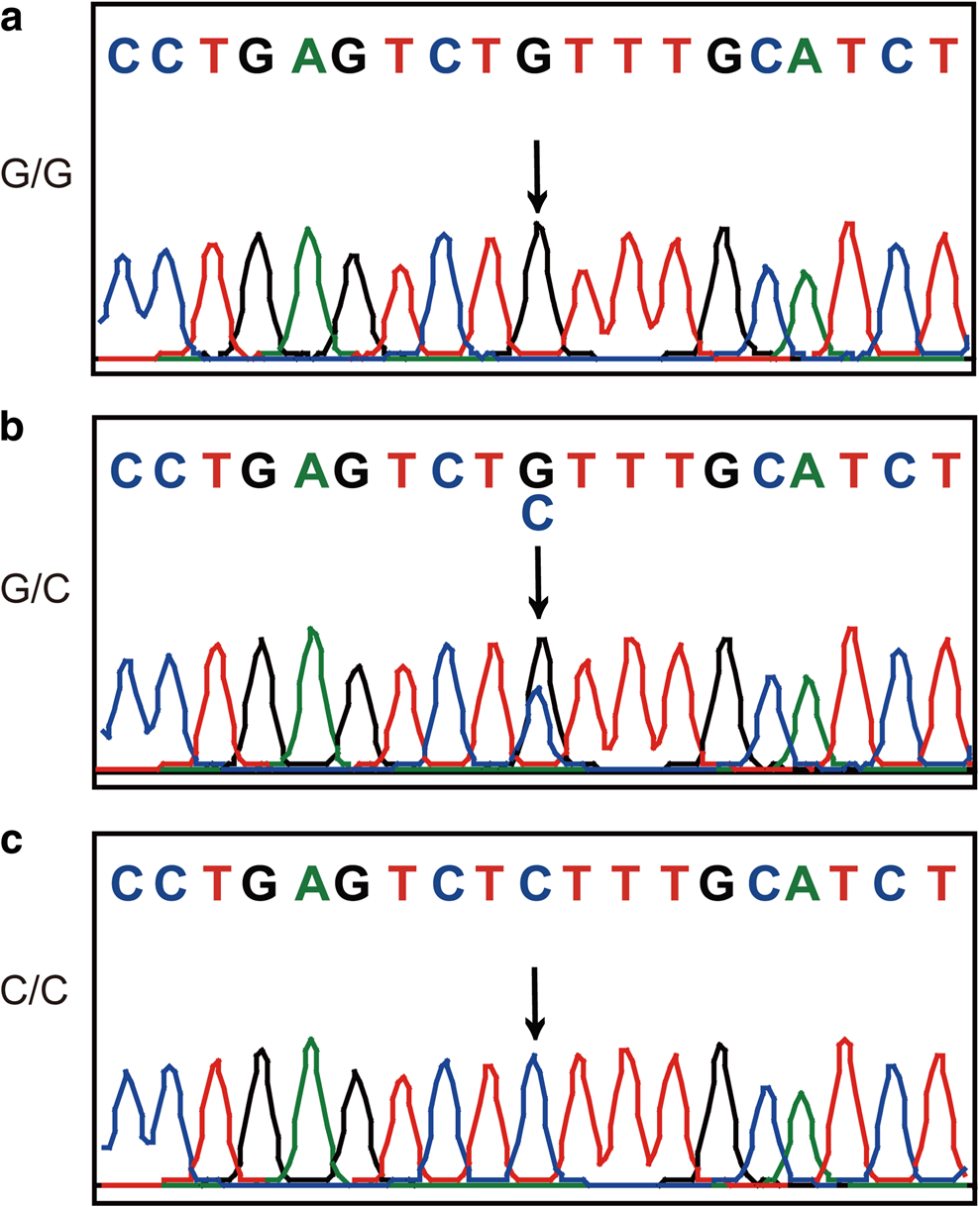
Representative chromograph of rs531564 sequence in each indicated genotype

The genotypes of rs531564 were in HWE in the control groups, both separately from Beijing or Gansu, and the combined control group (*P* = 0.16, *P* = 0.78, and *P* = 0.51, respectively) (Fig. 3). To explore the correlation between rs531564 and CHD susceptibility, both allelic and genotypic distribution were assessed. Additionally, various genetic models were applied with the adjustment for age and gender under unconditional logistic regression analyses (Table 3). The combined group data indicated that the C allele of rs531564 of *pri-miR-124* is associated with the decreased risk of CHD (OR = 0.72, 95% CI = 0.55–0.93, *P* = 0.01). In addition, similar results were obtained by analyzing the data with other models including the following: the codominant model (OR = 0.69, 95% CI = 0.51–0.93, *P* = 0.03), dominant model (OR = 0.67, 95% CI = 0.50–0.91, *P* = 0.01), overdominant model (OR = 0.70, 95% CI = 0.52–0.95, *P* = 0.02), and log-additive model (OR = 0.70, 95% CI = 0.53–0.91, *P* = 0.01) after adjustment. In the Beijing group, the genotype distribution of rs531564 was significantly different between CHD patients and healthy controls. Our finding indicated that minor C allele was significantly associated with the decreased risk of CHD in the Beijing group. This result was consistent among the analysis with different models including the allelic model (OR = 0.36, 95% CI = 0.21–0.63, *P* = 3e−04), the codominant model (OR = 0.30, 95% CI = 0.16–0.56, *P* = 4e−04), the dominant model (OR = 0.30, 95% CI = 0.16–0.56, *P* = 1e−04), the overdominant model (OR = 0.30, 95% CI = 0.16–0.57, *P* = 1e−04), and the log-additive model (OR = 0.33, 95% CI = 0.19–0.60, *P* = 1e−04). However, our findings did not support a significant correlation between rs531564 SNP and the risk of CHD in the Gansu group. In addition, it was noticed that the C allele frequency in the Gansu group was higher than that in the Beijing group in both the case-only group (0.15 and 0.08, respectively, *χ*2 = 8.38; *P* = 0.0038) and overall population (0.16 and 0.14, respectively, *χ*2 = 1.60; *P* = 0.21), while it is lower in the Gansu group than that in the Beijing group if the analysis was done in the control-only group (0.17 and 0.19, respectively, *χ*2 = 0.61; *P* = 0.43).

**Table 3:** Association between rs531564 polymorphism and CHD in two independent case-control studies

Genetic model	Pattern	Case	Control	*P* ^a^	OR (95% CI)^a^	P_*HWE*_	MAF (case/control)
Beijing group						0.16	
Allele	G	221	210	–	1.00		
	C	19	50	3e−04	0.36 (0.21–0.63)		0.08/0.19
Codominant	GG	102	82	–	1.00		
	GC	17	46	4e−04	0.30 (0.16–0.56)		
	CC	1	2	0.42	0.37 (0.03–4.22)		
Dominant	GC+CC/GG	18/102	48/82	1e−04	0.30 (0.16–0.56)		
Recessive	GG+GC/CC	119/1	128/2	0.56	0.50 (0.04–5.61)		
Overdominant	GC/GG+CC	17/103	46/84	1e−04	0.30 (0.16–0.57)		
Log-additive	GG/GC/CC	102/17/1	82/46/2	1e−04	0.33 (0.19–0.60)		
Gansu group						0.78	
Allele	G	528	531	–	1.00		
	C	96	109	0.427	0.89 (0.66–1.20)		0.15/0.17
Codominant	GG	222	221	–	1.00		
	GC	84	89	0.49	0.89 (0.63–1.28)		
	CC	6	10	0.32	0.57 (0.20–1.60)		
Dominant	GC+CC/GG	90/222	99/221	0.40	0.86 (0.61–1.22)		
Recessive	GG+GC/CC	306/6	310/10	0.30	0.58 (0.21–1.65)		
Overdominant	GC/GG+CC	84/228	89/231	0.62	0.91 (0.64–1.30)		
Log-additive	GG/GC/CC	222/84/6	221/89/10	0.29	0.85 (0.62–1.15)		
Combined group						0.51	
Allele	G	749	741	–	1.00		
	C	115	159	0.01	0.72 (0.55–0.93)		0.13/0.18
Codominant	GG	324	303	–	1.00		
	GC	101	135	0.03	0.69 (0.51–0.93)		
	CC	7	12	0.21	0.54 (0.21–1.40)		
Dominant	GC+CC/GG	108/324	147/303	0.01	0.67 (0.50–0.91)		
Recessive	GG+GC/CC	425/7	438/12	0.28	0.60 (0.23–1.54)		
Overdominant	GC/GG+CC	101/331	135/315	0.02	0.70 (0.52–0.95)		
Log-additive	GG/GC/CC	324/101/7	303/135/12	0.01	0.70 (0.53–0.91)		

*OR* odds ratio, *CI* confidence interval, *HWE* Hardy-Weinberg equilibrium, *MAF* minor allele frequency

P_*HWE*_, *P* value for HWE test in controls

^a^Adjusted for age and gender in the codominant, dominant, recessive, overdominant, and log-additive models

Significant associations with *P* value less than 0.05 were shown in boldface

It was shown in the stratification analysis of subtypes of CHD that rs531564 was significantly associated with isolated CHD (OR = 0.40, 95% CI = 0.19–0.86, *P* = 0.01 in dominant model after adjusted by gender and age) and complex CHD (OR = 0.21, 95% CI = 0.09–0.51, *P* = 1e−04 in dominant model after adjusted by gender and age) in the Beijing group. More specifically, the significant association of rs531564 was found in TOF cases (OR = 0.18, 95% CI = 0.04–0.82, *P* = 0.01 in dominant model after adjusted by gender and age) in the Beijing group (Table 4). There was no statistical correlation between rs531564 SNP and any subtypes of CHD in the Gansu group.

**Table 4:** Stratified analysis of rs531564 by CHD subtypes in the dominant model (GC+CC/GG)

	Beijing group		Gansu group		Combined group	
CHD classification^a^						
Isolated CHD	0.01	0.40 (0.19–0.86)	0.49	0.87 (0.60–1.28)	0.12	0.77 (0.55–1.07)
Complex CHD	1e−04	0.21 (0.09–0.51)	0.45	0.82 (0.48–1.40)	4e−03	0.52 (0.33–0.82)
Detailed CHD phenotypes						
ASD	0.03	0.16 (0.02–1.26)	0.49	0.83 (0.49–1.41)	0.19	0.72 (0.45–1.18)
VSD	0.06	0.48 (0.22–1.07)	0.77	0.94 (0.60–1.47)	0.29	0.81 (0.55–1.19)
TOF	0.01	0.18 (0.04–0.82)	0.38	0.66 (0.26–1.70)	0.03	0.47 (0.22–0.99)

Adjusted for age and gender

^a^Isolated CHD including ASD and VSD. Complex CHD including TOF, AVSD, TOF + ASD, ASD + PDA, VSD + PDA, ASD + PS, and VSD + PS

*CHD* congenital heart disease, *ASD* atrial septal defect, *VSD* ventricular septal defect, *TOF* tetralogy of Fallot, *AVSD* atrioventricular septal defect, *PDA* patent ductus arteriosus, *PS* pulmonary stenosis, *SD* standard deviation

Significant associations with *P* value less than 0.05 were shown in boldface

In summary, our results suggested that the rs531564 SNP of *pri-miR-124* correlated with the risk of CHD in the Beijing group.

## Discussion

In the present study, two gene expression profiles (GSE26125 and GSE35776) of non-syndromic TOF, of which the main pathological feature is chronic hypoxemia, were used to reveal the underlying molecular mechanisms in cardiomyocytes under hypoxic condition. A total of 330 DEGs were identified in non-syndromic TOF samples, including 318 upregulated genes and 12 downregulated genes. Functional and pathway enrichment analysis in DEGs revealed that genes involved in metabolic pathways and oxidative phosphorylation were significantly enriched. Besides the oxidative phosphorylation gene set, the GSEA found that the gene sets related to hypoxia, glycolysis, and fatty acid metabolism were enriched in non-syndromic TOF. In addition, the oxidative phosphorylation gene set was seen to be more statistically significant enriched than the glycolysis gene set. The above-mentioned results indicated that glycolysis and oxidative phosphorylation were upregulated in cardiomyocytes of non-syndromic TOF and the upregulated oxidative phosphorylation may predominantly relate to the maintenance of energy production. For TOF patients, anatomic defects lead to oxygen deficiency from the decreased pulmonary blood flow. Traditionally, cardiomyocyte energy metabolism shifts from oxidative phosphorylation to glycolysis to maintain ATP levels under the stressful hypoxic conditions (Azzouzi et al. 2015; Balaban 2012). However, ATP is produced solely from glycolysis only if the oxygen concentration limits the function of oxidative phosphorylation, which is the case in severe hypoxia (Hollinshead and Tennant 2016). The changes of metabolism found in non-syndromic TOF patients may be attributed to the effect of chronic mild hypoxia on oxidative phosphorylation.

MiRNAs is a class of non-coding short RNAs with 19 to 24 nucleotides and regulates gene expression by binding to the 3′ untranslated region of target mRNA at the post-transcriptional level (Azzouzi et al. 2015). Therefore, miRNAs influence various cellular processes such as metabolism, differentiation, proliferation, and apoptosis. As part of this study, we also screened DEMIs in the TOF-related miRNA profile GSE35490. Finally, 29 pairs of miRNA-mRNA, including 10 miRNAs and 28 mRNAs, were identified by integrated analysis of three target gene prediction databases. We found that the downregulated miR-124 is likely to be associated with metabolic pathways in addition to oxidative phosphorylation by modulating the potential target genes (*AK2*, *AK3*, *ACAA2*) in key modules of the PPI network. Besides, seven potential target genes (*SDC4*, *PGM1*, *NR3C1*, *GPC1*, *AK3*, *GLRX*, and *RBCK1*) of miR-124 were positively correlated with hypoxia and glycolysis gene sets in non-syndromic TOF. The entire above-mentioned miR-124 target genes have been considered to be related to metabolism (Garcia-Esparcia et al. 2015; Laforet et al. 2017). In addition, previous studies have suggested that hypoxia induces miR-124 downregulation which promotes prostate cancer cell survival, leads to chondrogenesis, and promotes proliferation of pulmonary artery smooth muscle cells. These functions are mediated by the regulation of *PIM1*, *NFATc1*, and *GRB2*, respectively (Gong et al. 2017; Gu et al. 2016; Li et al. 2017). The downregulated miR-124 also exhibits a cardio-protective effect by attenuating endoplasmic reticulum stress (Bao et al. 2017). Therefore, it is reasonable that miR-124 may play an important role in regulating myocardial metabolism in non-syndromic TOF. Given that metabolism, especially folate metabolism, has an important influence on cardiac development and gene expression specificity at different stages of organ development, further investigation is needed to unveil the relationship between miR-124 and folate metabolism or heart development under hypoxic conditions (Blom and Smulders 2011; Cripps and Olson 2002).

It is critical for healthy heart development in a hypoxic intrauterine environment during early fetal life, but long-term exposure to the hypoxic environment may lead to fetal intrauterine growth restriction (Bae et al. 2003; Gagnon 2003; Herrera et al. 2016; Yue and Tomanek 1999). Chronic intrauterine hypoxia also affects heart development at some stages, and possible molecular mechanisms include that chronic fetal hypoxia alters cardiac gene expression, accelerates pre-existing cardiomyocytes exit the cell cycle, increases myocyte apoptosis, and reduces in the number of cardiomyocytes (Botting et al. 2014; Osterman et al. 2015; Zhang 2005). Furthermore, studies have shown that the incidence rate and the severity of CHD are higher in high-altitude areas. Hypoxic environment was considered as a risk factor of congenital heart disease in high-altitude areas (Hasan 2016; Miao et al. 1988; Zheng et al. 2013). As mentioned above, miR-124 is involved in the regulation of cardiomyocyte metabolism under hypoxic conditions. Moreover, it is widely reported that the G allele of rs531564, in *pri-miR-124*, is associated with increased mature miR-124 expression (Chen et al. 2018; Li et al. 2017; Zou et al. 2017). In this study, we evaluated the association between the rs531564 polymorphism of *pri-miR-124* and CHD in two independent groups of Chinese patients who were from Beijing (with altitudes ranging from 28 to 96 m) or Gansu (with altitudes ranging from 1085 to 2690 m). Our findings revealed that the C allele of rs531564 of *pri-miR-124* is associated with the decreased risk of CHD, especially complex CHD, in the group from Beijing, but not in the Gansu group. The correlation between the different geographical areas and CHD risk is different for the rs531564 SNP in this study. It seems to be consistent with the report that the etiology of sporadic, non-syndromic CHD involves the interaction of multiple genetic and environmental factors (Hinton 2013). The decrease risk of CHD risk associated with the C allele of rs531564 may be partly explained by the lower expression level of mature miR-124 than that of the G allele. Low levels of miR-124 expression contribute to the maintenance of cardiomyocyte metabolism under hypoxic conditions caused by placental insufficiency or hypoxic environmental exposure. Additionally, we noticed that although there was no statistical correlation between the C allele and CHD in the tested patients from Gansu, where the altitude is high, the C allele frequency in the Gansu group was higher than that in the Beijing group in the case-only group or overall population. These data indicated that the higher frequency of the C allele in the Gansu group is likely to be related to environmental selection pressure (Verhulst and Neale 2016). Further investigation is needed to explore whether this SNP is associated with positive selection for high-altitude hypoxic adaptation.

In summary, two mRNA profiles as well as a miRNA profile of non-syndromic TOF provided evidence that miR-124 is involved in the regulation of cardiomyocyte metabolism under hypoxic conditions. This study also revealed a significant correlation between the rs531564 SNP of *pri-miR-124* and the risk of CHD in population from area with low altitude, but not in population from areas with high altitude. Our results provide new insights into the etiology of sporadic, non-syndromic CHD. However, given the limitations of this study in terms of sample size, narrow range of the altitude of the involved area and the single ethnicity, more comprehensive studies with larger sample size, different regions, and ethnic groups are needed to confirm our findings.

## Electronic supplementary material

Fig. S1 Identification of DEGs and DEMIs. **a, b, c** Volcano plot of GSE26125, GSE35776, and GSE35490 under the thresholds of |log2(FC)| > 1.00 and P < 0.05; **d, e** Venn diagram illustrating the overlapped upregulated DEGs and downregulated miRNA potential targets, downregulated DEGs and upregulated miRNA potential targets. (PNG 149 kb)

High resolution image (TIF 4354 kb)

Table S1 (XLS 121 kb)

Table S2 (DOC 41 kb)

Table S3 (DOC 33 kb)

Table S4 (DOC 36 kb)
