# Supplementary figures and images for: Correlation between pri-miR-124 (rs531564) polymorphism and congenital heart disease susceptibility in Chinese population at two different altitudes: a case-control and in silico study
Source: Environ Sci Pollut Res Int. 2019 May 29;26(21):21983–92. doi: 10.1007/s11356-019-05350-4 (PMC6657426; doi:10.1007/s11356-019-05350-4)

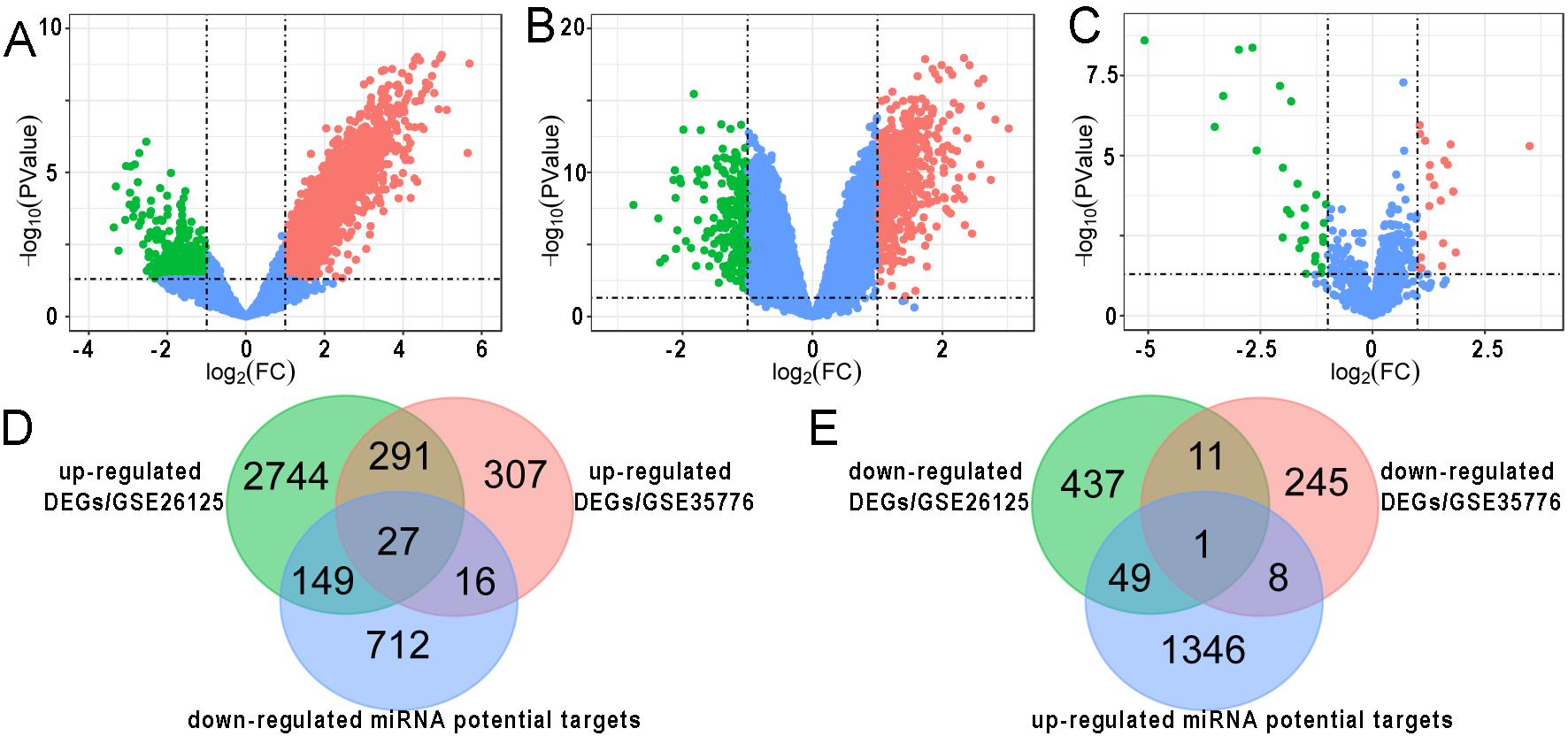

Supplement: Supplementary file 1 — Identification of DEGs and DEMIs. a, b, c Volcano plot of GSE26125, GSE35776, and GSE35490 under the thresholds of |log2(FC)| > 1.00 and P < 0.05; d, e Venn diagram illustrating the overlapped upregulated DEGs and downregulated miRNA potential targets, downregulated DEGs and upregulated miRNA potential targets. (PNG 149 kb) [file 11356_2019_5350_Fig4_ESM.png]

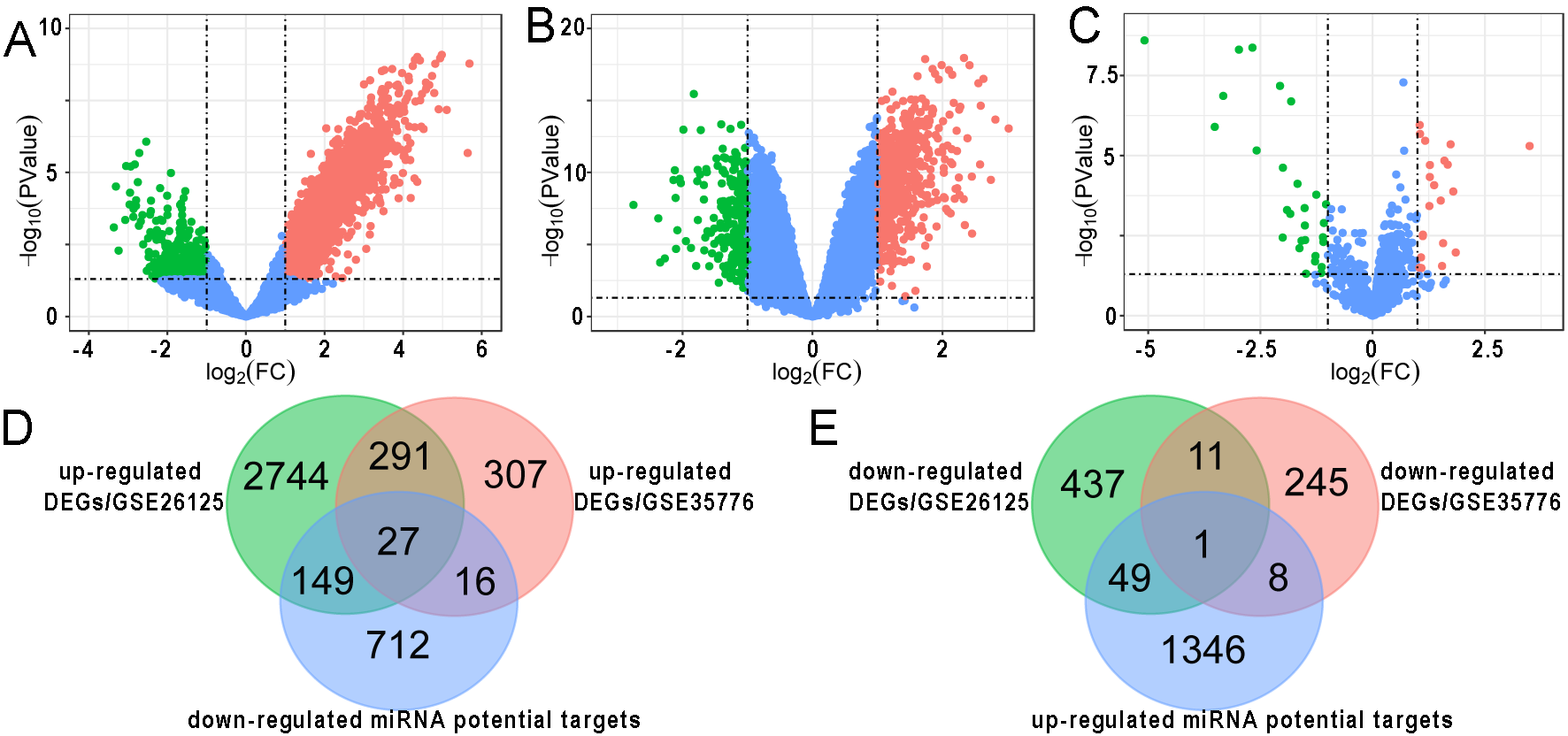

Supplement: Supplementary file 2 — High resolution image (TIF 4354 kb) [file 11356_2019_5350_MOESM1_ESM.tif]
