# Supplementary material for: Correlation between pri-miR-124 (rs531564) polymorphism and congenital heart disease susceptibility in Chinese population at two different altitudes: a case-control and in silico study
Source: Environ Sci Pollut Res Int. 2019 May 29;26(21):21983–92. doi: 10.1007/s11356-019-05350-4 (PMC6657426; doi:10.1007/s11356-019-05350-4)
Supplement: Supplementary file 4 — (DOC 41 kb) [file 11356_2019_5350_MOESM3_ESM.doc]

Table S2 Gene ontology biological process term and KEGG pathway enrichment analysis of up-regulated genes in non-syndromic TOF (Top 10)

| Term | ID | *P* | Cor. *P* |
| --- | --- | --- | --- |
| GO Term_BP | GO:0009987 Cellular process | 2.09e-23 | 5.64e-21 |
|  | GO:0044699 Single-organism process | 2.09e-26 | 8.24e-24 |
|  | GO:0008152 Metabolic process | 1.14e-20 | 2.44e-18 |
|  | GO:0071704 Organic substance metabolic process | 1.36e-16 | 2.17e-14 |
|  | GO:0044237 Cellular metabolic process | 3.07e-17 | 5.07e-15 |
|  | GO:0044238 Primary metabolic process | 7.80e-14 | 1.05e-11 |
|  | GO:0071840 Cellular component organization or biogenesis | 3.34e-12 | 4.07e-10 |
|  | GO:1901564 Organonitrogen compound metabolic process | 5.56e-19 | 9.82e-17 |
|  | GO:0044281 Small molecule metabolic process | 5.53e-19 | 9.82e-17 |
|  | GO:0055114 Oxidation-reduction process | 1.54e-20 | 3.17e-18 |
| KEGG pathway | hsa01100 Metabolic pathways | 7.96e-14 | 1.86e-11 |
|  | hsa00190 Oxidative phosphorylation | 2.27e-09 | 2.65e-07 |
|  | hsa03050 Proteasome | 2.99e-07 | 1.75e-05 |
|  | hsa00280 Valine, leucine and isoleucine degradation | 5.70e-05 | 0.002 |
|  | hsa05416 Viral myocarditis | 0.001 | 0.004 |
|  | hsa00072 Synthesis and degradation of ketone bodies | 0.001 | 0.012 |
|  | hsa04931 Insulin resistance | 0.001 | 0.014 |
|  | hsa01200 Carbon metabolism | 0.001 | 0.014 |
|  | hsa00640 Propanoate metabolism | 0.001 | 0.017 |
|  | hsa00350 Tyrosine metabolism | 0.001 | 0.022 |

Cor. *P*: Corrected *P*-Value
