# Supplementary material for: Correlation between pri-miR-124 (rs531564) polymorphism and congenital heart disease susceptibility in Chinese population at two different altitudes: a case-control and in silico study
Source: Environ Sci Pollut Res Int. 2019 May 29;26(21):21983–92. doi: 10.1007/s11356-019-05350-4 (PMC6657426; doi:10.1007/s11356-019-05350-4)
Supplement: Supplementary file 5 — (DOC 33 kb) [file 11356_2019_5350_MOESM4_ESM.doc]

Table S3 KEGG pathway enrichment analysis of gene modules and DEMI-DEG regulatory relationships in gene modules

| Module 1, Score 9.300 |  |  |  |  |
| --- | --- | --- | --- | --- |
| KEGG pathway | *P* | Cor. *P* | Node IDs | DEMI(DEG) |
| hsa03050 Proteasome | 2.36e-19 | 1.89e-18 | *ASB12, COPS2, RNF14, ASB11, PSME1, PSMA4, ASB5, ASB8, PSMA2, FBXO32, POMP, PSMA3, PSMD10, PSMB5, ANAPC16, PSMC4, ANAPC13, PSMA1, RNF146, UBR1, PSMG2* | miR-297 (*ASB11*),  miR-595 (*PSMA2*),  miR-1253 (*RNF14*),  miR-1248 (*RNF146*) |
| Module 2, Score 8.750 |  |  |  |  |
| KEGG pathway | *P* | Cor. *P* | Node IDs | DEMI(DEG) |
| hsa00190 Oxidative phosphorylation | 4.22e-18 | 2.95e-17 | *COX5B, ATP5L, NDUFB5, NDUFA12, PMPCB, UQCRB*, etc. |  |
| hsa01100 Metabolic pathways | 3.14e-10 | 3.66e-10 |
| hsa04260 Cardiac muscle contraction | 0.001 | 0.001 |

Cor. *P*: Corrected *P*-Value
