# Supplementary material for: Correlation between pri-miR-124 (rs531564) polymorphism and congenital heart disease susceptibility in Chinese population at two different altitudes: a case-control and in silico study
Source: Environ Sci Pollut Res Int. 2019 May 29;26(21):21983–92. doi: 10.1007/s11356-019-05350-4 (PMC6657426; doi:10.1007/s11356-019-05350-4)
Supplement: Supplementary file 6 — (DOC 36 kb) [file 11356_2019_5350_MOESM5_ESM.doc]

Table S4 Overlapped GSEA results between GSE26125 and GSE35776

| Name | Size | NES | *P* | FDR |
| --- | --- | --- | --- | --- |
| HALLMARK_OXIDATIVE PHOSPHORYLATION | 172/163 | 2.539/3.911 | 0/0 | 0/0 |
| HALLMARK_MYC TARGETS V1 | 160/148 | 1.938/2.593 | 0/0 | 4.79e-04/0 |
| HALLMARK_FATTY ACID METABOLISM | 113/135 | 1.900/3.009 | 0/0 | 3.19e-04/0 |
| HALLMARK_ADIPOGENESIS | 139/178 | 1.782/2.980 | 0/0 | 7.51e-04/0 |
| HALLMARK_MYOGENESIS | 160/172 | 1.690/1.395 | 0/0.026 | 0.004/0.067 |
| HALLMARK_MTORC1_SIGNALING | 150/175 | 1.669/2.329 | 0/0 | 0.004/0 |
| HALLMARK_DNA REPAIR | 101/128 | 1.666/1.881 | 0/0 | 0.004/0.002 |
| HALLMARK_PEROXISOME | 67/91 | 1.539/1.677 | 0.005/0 | 0.020/0.012 |
| HALLMARK_ANDROGEN RESPONSE | 65/86 | 1.442/1.451 | 0.020/0.043 | 0.047/0.046 |
| HALLMARK_UNFOLDED PROTEIN RESPONSE | 82/98 | 1.472/2.057 | 0.011/0 | 0.040/0 |
| HALLMARK_PROTEIN SECRETION | 67/86 | 1.373/3.059 | 0.038/0 | 0.092/0 |
| HALLMARK_GLYCOLYSIS | 136/179 | 1.292/1.635 | 0.027/0 | 0.181/0.013 |
| HALLMARK_HYPOXIA | 141/181 | 1.272/1.298 | 0.045/0.037 | 0.207/0.126 |

NES, normalized enrichment score. FDR, false discovery rate.
